# Characterization of a lactate metabolism-related signature for evaluation of immune features and prediction prognosis in glioma

**DOI:** 10.3389/fneur.2022.1064349

**Published:** 2023-01-09

**Authors:** Zhiqiang Wu, Jing Wang, Yanan Li, Jianmin Liu, Zijian Kang, Wangjun Yan

**Affiliations:** ^1^Department of Musculoskeletal Surgery, Shanghai Cancer Center, Fudan University, Shanghai, China; ^2^Department of Oncology, Shanghai Medical College, Fudan University, Shanghai, China; ^3^Neurovascular Center, Changhai Hospital, Naval Medical University, Shanghai, China; ^4^Department of Rheumatology and Immunology, Second Affiliated Hospital of Naval Medical University, Shanghai, China

**Keywords:** glioma, lactate metabolism, immune infiltration, prognostic model, CGGA

## Abstract

**Background:**

Glioma is one of the most typical tumors in the central nervous system with a poor prognosis, and the optimal management strategy remains controversial. Lactate in the tumor microenvironment is known to promote cancer progression, but its impact on clinical outcomes of glioma is largely unknown.

**Methods:**

Glioma RNA-seq data were obtained from TCGA and GCGA databases. Lactate metabolism genes (LMGs) were then evaluated to construct an LMG model in glioma using Cox and LASSO regression. Immune cell infiltration, immune checkpoint gene expression, enriched pathways, genetic alteration, and drug sensitivity were compared within the risk subgroups. Based on the risk score and clinicopathological features, a nomogram was developed to predict prognosis in patients with glioma.

**Results:**

Five genes (LDHA, LDHB, MRS2, SL16A1, and SL25A12) showed a good prognostic value and were used to construct an LMG-based risk score. This risk score was shown as an independent prognostic factor with good predictive power in both training and validation cohorts (*p* < 0.001). The LMG signature was found to be correlated with the expression of immune checkpoint genes and immune infiltration and could shape the tumor microenvironment. Genetic alteration, dysregulated metabolism, and tumorigenesis pathways could be the underlying contributing factors that affect LMG risk stratification. The patients with glioma in the LMG high-risk group showed high sensitivity to EGFR inhibitors. In addition, our nomogram model could effectively predict overall survival with an area under the curve value of 0.894.

**Conclusion:**

We explored the characteristics of LMGs in glioma and proposed an LMG-based signature. This prognostic model could predict the survival of patients with glioma and help clinical oncologists plan more individualized and effective therapeutic regimens.

## 1. Introduction

Glioma is one of the most typical tumors in the central nervous system, comprising approximately 80% of primary brain tumors (1). Glioma can be classified into four grades: grades II and III are defined as diffuse lower-grade gliomas, and grade IV glioma is also termed glioblastoma (2). Globally, glioma is mostly treated by surgery followed by postoperative radiotherapy and chemotherapy (3). Despite considerable advances in the development of treatments, the prognosis of patients with glioma remains poor, and the optimal management strategy remains controversial. The 5-year survival rate for patients with high-grade glioma is only ~5% (1). Although low-grade gliomas have a better prognosis than glioblastomas, they are more likely to recur and transit into high-grade gliomas (4, 5). Thus, identifying new and reliable biomarkers of glioma development and progression is urgently needed to guide clinical management and find potential targets for patients with glioma.

The presence of lactate in human tumors has long been neglected. It is now rediscovered as an important carbon source for cellular metabolism and as a signaling molecule in cancerous tissues (6). Lactate in the tumor microenvironment (TME) promotes cancer progression by creating an active niche that can shape tumor pathogenesis and evolution (7). It also reserves the acidic phenotype and increases tumor progression by modulating the TME, including cell invasion, angiogenesis, survival signaling, metastasis development, and immune surveillance escape (6). Extracellular acidosis suppresses T cell-mediated immunity, thus reducing cytolytic activity and cytokine production. Numerous studies have demonstrated that neutralization of tumor acidity in immunotherapy can improve antitumor responses (8). Extracellular lactate levels can be sensed by several cell types, including cancer cells, T cells, NK cells, dendritic cells, and macrophages, triggering intracellular signaling and strongly impacting cell behaviors and functions in the TME (9–12). Therefore, the identification of how lactate metabolism regulators mediate the TME may help improve the survival prognosis of patients with glioma.

In this study, we analyzed the expression and characterized the genomic dysregulation of lactate metabolism genes (LMGs) in glioma and determined that LMGs were associated with the tumorigenesis and prognosis of glioma. In addition, we further constructed a prediction model and revealed a high efficacy for prognosis prediction. We also explored the association between LMGs and the immune microenvironment using the constructed signature. Our study aimed to comprehensively assess the correlation of LMGs with the prognosis and immune microenvironment in glioma.

## 2. Methods

### 2.1. Data collection

The gene expression data and clinical characteristics of patients with glioma in the training set were obtained from the Chinese Glioma Genome Altas (CGGA)-RNA-seq dataset (693) (http://www.cgga.org.cn/). The Cancer Genome Atlas (TCGA) dataset (https://portal.gdc.cancer.gov) and the CGGA RNA-seq dataset (325) were used as two independent datasets. The included patients and detailed metabolism-related genes are listed in Supplementary Table 1. Genetic mutation profiles were obtained from the cBioPortal dataset (http://www.cbioportal.org/). Notably, 26 LMGs were downloaded from the Molecular Signature Database version 7.0 (MSigDB) (http://www.broad.mit.edu/gsea/msigdb/). The representative immunohistochemical (IHC) staining images of LMGs were obtained from the Human Protein Atlas (HPA) database (https://www.proteinatlas.org/).

### 2.2. Clinical sample collection

To determine the expression of LMGs between tumor tissues and paracancerous tissues of glioma, six samples from Changhai Hospital were collected for quantitative real-time polymerase chain reaction (qRT-PCR). The primer of the six LMGs is shown in Supplementary Table 2. Ethical approval was granted by the Ethics Committee of Shanghai Changhai Hospital (Ethics approval number: CHEC2020-164). All participants were fully informed of the research and provided the informed consent forms.

### 2.3. Protein–protein interaction network analysis

The STRING functional protein association network (https://string-db.org) (13) was used to construct the interaction network. The input genes consisted of LMGs to a high confidence (0.4) of active interaction. Moreover, functional enrichment analysis was carried out on the LMGs.

### 2.4. Construction and evaluation of the LMG-based prognostic risk score model

Univariate cox regression analysis on the LMGs in the training cohort was performed to identify the association between the expression levels of the genes and overall survival (OS) time using the survival package. Significant genes with a *p*-value of < 0.05 identified by univariate cox regression were further screened by least absolute shrinkage and selection operator (LASSO) cox regression. Then, six optimal LMGs were used to construct a prognostic risk score model using the following formula: risk score = ∑Coefi·Expi (Coef is the regression coefficient calculated by the LASSO). According to the calculated prognostic risk score, all patients were divided into a high-risk and a low-risk group. Kaplan-Meier survival curve and time-dependent receiver operating characteristic (ROC) curve were used to evaluate the prognostic performance of the constructed risk model.

### 2.5. Immune cell infiltration analysis

The abundance of immune cells in tumor tissue was calculated using the Cibersort algorithm (http://CIBERSORT.stanford.edu/), which transformed the normalized gene expression matrix into the composition of infiltrating immune cells. The LM22 signature matrix defined 22 types of immune cell components and was used as a reference expression signature with 1,000 permutations.

### 2.6. Gene set variation analysis and gene set enrichment analysis

Hallmark gene sets and curated gene sets were downloaded from the MsigDB dataset. Gene set variation analysis (GSVA) was utilized to evaluate the relative enrichment of the 50 hallmark pathways across samples using a non-parametric approach (14). The correlation between the LMG score and the 50 cancer Hallmark Pathway score was calculated by Spearman analysis and was visualized in a heatmap. Gene set enrichment analysis (GSEA) for the curated gene sets was performed using the R package ClusterProfiler (15) based on the differentially expressed genes. Significant gene sets were defined by false discovery rate (FDR) < 0.05 and normalized enrichment score (NES) > 2. The results were visualized by R package enrichplot.

### 2.7. Nomogram construction and verification

The clinical features and LMG signature were used to develop the nomogram for glioma prognostic prediction using the “rms” package (16). Calibration curves and ROC curves for 1, 2, 3, and 5 years were used to assess the accuracy and discrimination ability of the nomogram.

### 2.8. Single nucleotide variation and copy number variation analysis

The single nucleotide variation (SNV) and copy number variation (CNV) data of patients with glioma were obtained from the TCGA dataset. In brief, the TCGAbiolinks R package was used to download the SNV and CNV data from the TCGA database. The maftools R package was utilized to analyze SNV data and visualize the mutation waterfall plots. CNV data from the two different risk groups were analyzed using the GISTIC2.0 module on GenePattern (https://cloud.genepattern.org/gp/pages/index.jsf) with default parameters.

### 2.9. Drug sensitivity analysis

Genomics of Drug Sensitivity in Cancer (GDSC; https://www.cancerrxgene.org/) (17) is the largest public pharmacogenomics database, which is used for exploring molecular cancer therapy and mutation. The R package pRRophetic (18) was used to screen chemotherapeutic agents in the high- and low-risk groups.

### 2.10. RNA isolation, reverse transcription, and qRT-PCR

First, RNA from glioma and paracancerous tissues was extracted using Trizol (Invitrogen, USA) extraction reagents. Isolated RNA was converted into cDNA *via* a cDNA reverse transcription kit (TaKaRa, Cat: RR047A, Japan). Following this, the relative expression of cDNA was visualized using the SYBR qPCR Master Mix (Takara, Cat: RR820A, Japan) on a Real-Time PCR system (Applied Biosystems, Foster City, USA). β-Actin was used as a reference gene, and relative gene expression was quantified using the formula: 2–^ΔΔ^Ct.

### 2.11. Statistical analysis

Statistical analyses were mainly performed using R version 3.6.2. Kaplan–Meier and log-rank analyses were performed to evaluate the survival differences between different groups of patients. Student's *t*-test and one-way ANOVA analysis were used to estimate the differences between two groups and more than two groups. The correlation analysis was calculated using the “Spearman” method. Two-sided *p* < 0.05 was regarded as statistically significant.

## 3. Results

### 3.1. Correlation between LMG expression and the clinical characteristics in glioma

**Figure 1:**
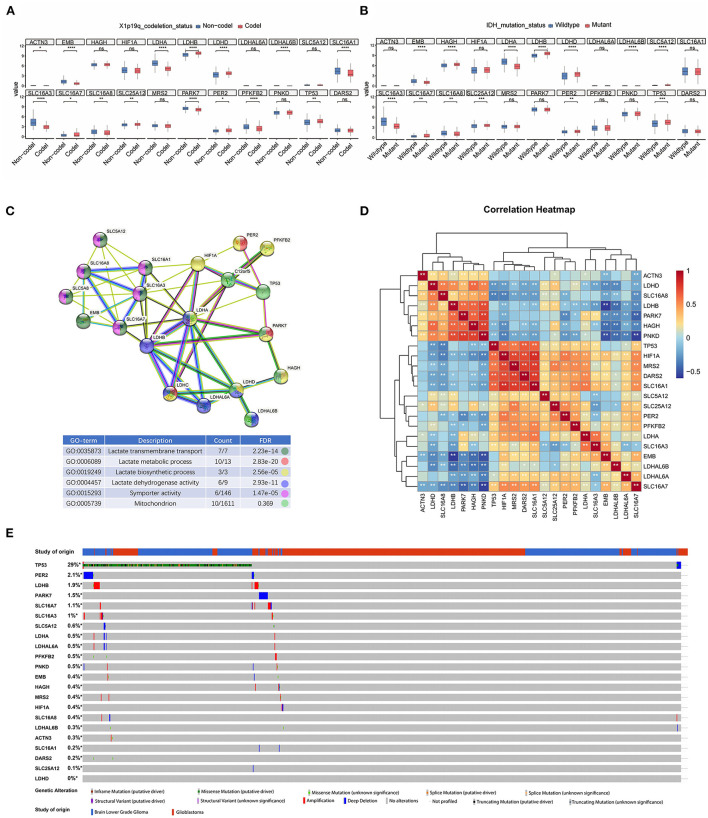
Transcriptome and genetic alteration profiles of lactic acid metabolism-related genes (LMGs) in glioma. **(A)** Box plot showing the expression of LMGs in patients with X1p9q codel and non-Codel glioma. **(B)** Box plot showing the expression of LMGs in IDH mutate and patients with wild glioma. **(C)** Protein–protein interaction (PPI) network and gene ontology enrichment analysis of the LMGs. **(D)** Heatmap showing the correlation of the LMGs in glioma. **(E)** Genetic alteration profiles of the LMGs from patients with glioma inn TCGA dataset. *p < 0.05, **p < 0.01, ***p < 0.001, ****p < 0.0001.

To identify the significance of lactate metabolism in glioma, we collected 26 LMGs from the MSigDB dataset and analyzed their expression in glioma with different clinical characteristics. It was found that the expression levels of LDHB, LDHD, SLC16A7, SLC25A12, PER2, and TP53 in 1p/19q Codel glioma were higher than those in non-Codel glioma. The expression levels of EMB, LDHA, SLC16A1, SLC16A3, SLC16A8, PARK7, and PFKFB2 were lower in 1p/19q Codel glioma than those in non-Codel glioma (Figure 1A). Furthermore, the expressions of HAGH, LDHB, LDHD, SLC16A7, SLC25A12, and TP53 were upregulated, and the expressions of EMB, LDHA, SLC16A3, and SLC16A8 were downregulated in isocitrate dehydrogenase (IDH) wild-type glioma compared with mutant glioma (Figure 1B). To investigate the protein interactions between LMGs and their biological functions, we constructed the interactions between genes related to lactate metabolism and the enriched their gene ontology (GO) functions by these interactions from the STRING database. The results showed that there were interactions between LMGs and that LDHA, LDHB, and SLC16A3 had more interactions relative to other genes (Figure 1C). These genes were enriched in the lactate transmembrane transport, lactate metabolic process, and biosynthetic process (Figure 1C). Correlation analysis showed a strong positive correlation between some of the LMGs. For example, TP53, HIF1A, MRS2, DRAS2, and SLC16A1 were positively correlated, and LDHB, PARK7, HAGH, and PNKD were also positively correlated, suggesting that they may play a synergistic role in lactate metabolism (Figure 1D). We also examined the mutational panorama of LMGs and observed that the frequencies of mutation change with the LMGs were quite low in the TCGA cohort, except for TP53, which may imply that their transcriptional levels were relatively stable in performing biological functions (Figure 1E).

### 3.2. Prognostic significance of LMGs in glioma

**Figure 2:**
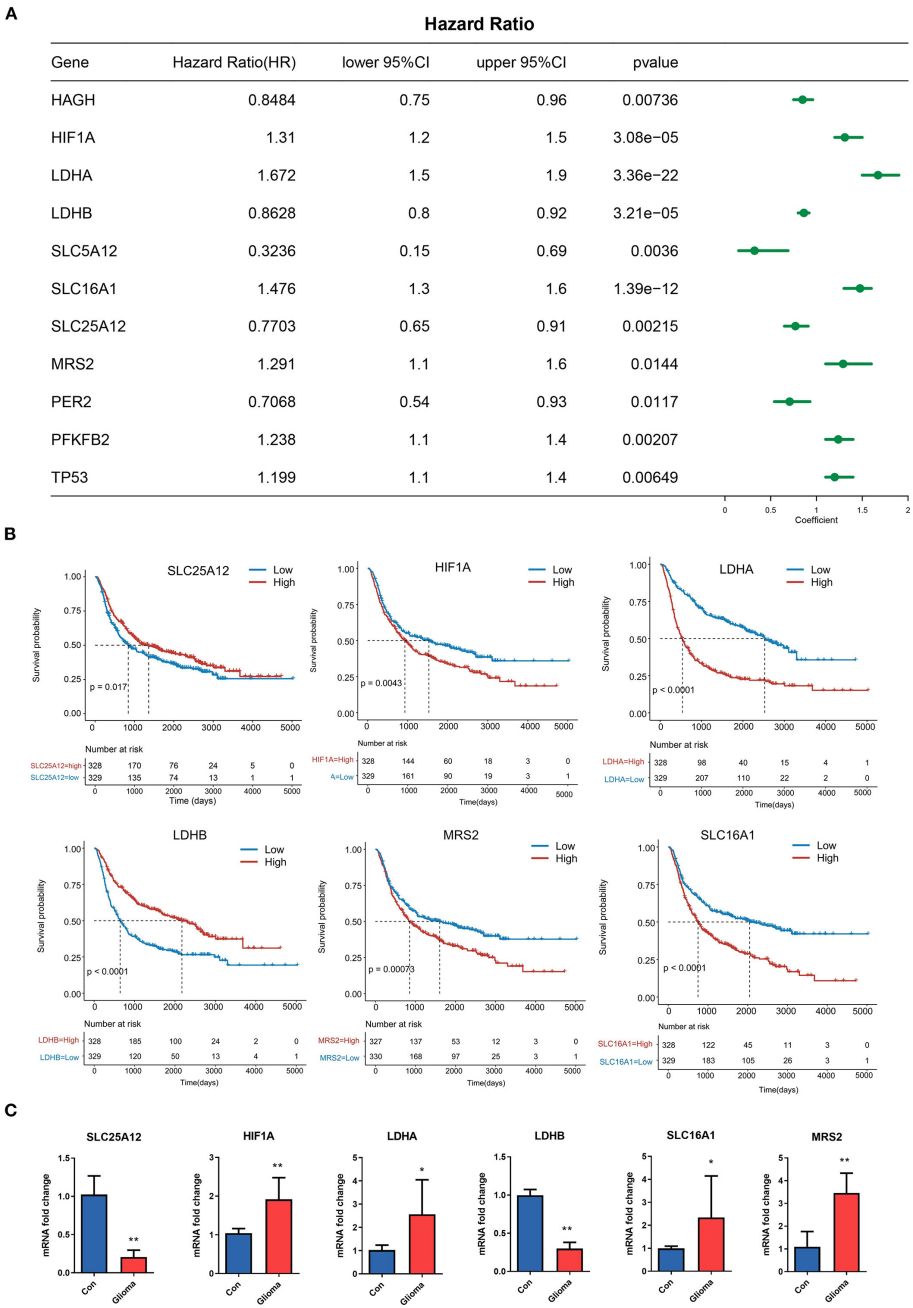
Prognostic significance of LMGs in patients with glioma. **(A)** Cox regression analysis showing the hazard ratios (HRs) for LMGs with 95% confidence intervals (CIs). **(B)** Kaplan–Meier curves for survival states of the representative genes identified by univariate cox regression. **(C)** Expression of six LMGs at the mRNA level by qRT-PCR. *p < 0.05, **p < 0.01.

To explore the prognostic significance of LMGs in glioma, we performed univariate cox proportional hazards regression analysis and evaluated the relationship between the gene expression value of LMGs and the OS status of patients with glioma. We found that 11 genes were significantly associated with OS time (*p* < 0.05, Figure 2A). Among the 11 genes, 6 genes (HIF1A, LDHA, SLC16A1, MRS2, PFKB2, and TP53) were identified as high-risk factors (hazard ratio >1) and 5 genes (HAGH, LDHB, SLE16A1, SLC25A12, and PER2) were identified as protective factors (hazard ratio < 1). These genes were subjected to Kaplan-Meier analysis, and the representative genes are shown in Figure 2B. Moreover, the IHC profiles generated from the HPA datasets also provided information about the location of LMGs and their expression status (Supplementary Figures S1A–L). We used qPCR to further validate the LMG expression. The results showed that glioma tissues have higher expression of LDHA, HLF1A, SLC16A1, and MRS2 and lower expression of LDHB and SLC25A12 compared with paracancerous tissues (Figure 2C). These results suggested that LMGs were correlated with outcomes in patients with glioma and might play a role in glioma progression.

### 3.3. A prognostic risk model constructed from the LMGs

**Figure 3:**
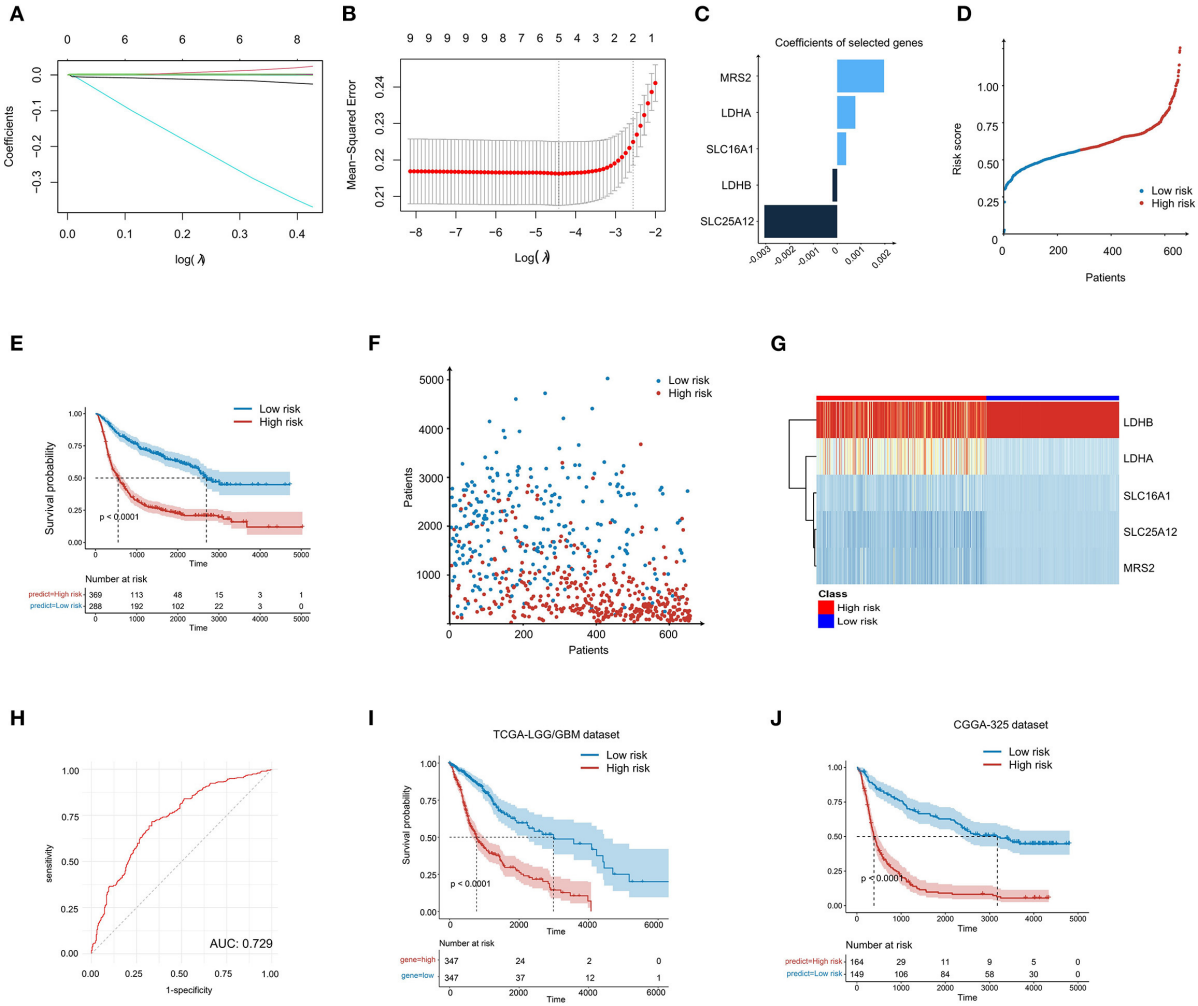
Construction of LMG-based risk model in patients with glioma. **(A)** LASSO coefficient profiling of the five LMGs. **(B)** Cross-validation for tuning parameter selection in the proportional hazards model. **(C)** The five genes' coefficients screened by lasso regression. **(D)** LMG signature distribution of patients with glioma in the CGGA-693 cohort. **(E)** Kaplan–Meier plot showing the overall survival (OS) for low- and high-LMG groups using the log-rank test. **(F)** Survival state distribution of patients with glioma in the CGGA-693 cohort. **(G)** Heatmap showing the expression level of five LMGs in the CGGA-693 cohort. **(H)** Time-dependent ROC analysis for the risk score in the CGGA-693 cohort. **(I, J)** Kaplan–Meier plot showing the OS for low- and high-risk LMG signature groups in the TCGA cohort and the CGGA-325 cohort.

Considering the prognostic value of some LMGs in glioma, we intended to construct a risk score model to evaluate the prognosis status of glioma samples more accurately. The 11 prognosis-related genes were further performed using the LASSO regression analysis to screen the most valuable predictive genes (Figures 3A, B). Five genes were selected with a *p* < 0.05 and the risk signature included MRS2, LDHA, SLC16A1, LDHB, and SLC25A12 (Figure 3C). Based on the risk score, we divided the patients with glioma into high-risk and low-risk groups (Figure 3D). It was found that the LMG signature could effectively differentiate cancer prognosis. Patients with glioma with high-risk scores had a shorter survival time and poorer prognosis as compared with those with low-risk scores (Figures 3E, F). In the high-risk group, the expression of MRS2, LDHA, and SLC6A1 was higher, and the expression of LDHB and SLC25A12 was lower than that in the low-risk group (Figure 3G). The risk score exhibited a high prognostic validity, with an area under curve (AUC) value of 0.729 (Figure 3H). To further validate the robustness of the LMG model, we utilized two independent sources of glioma to test the robustness of our model. The result of the Kaplan-Meier survival analysis showed that the LMG model was effective in both datasets (Figures 3I, J). The above results suggest that our LMG-based prognostic model could effectively predict glioma prognosis.

### 3.4. Prognostic value of the LMG signature for subgroups of clinical classifications

**Figure 4:**
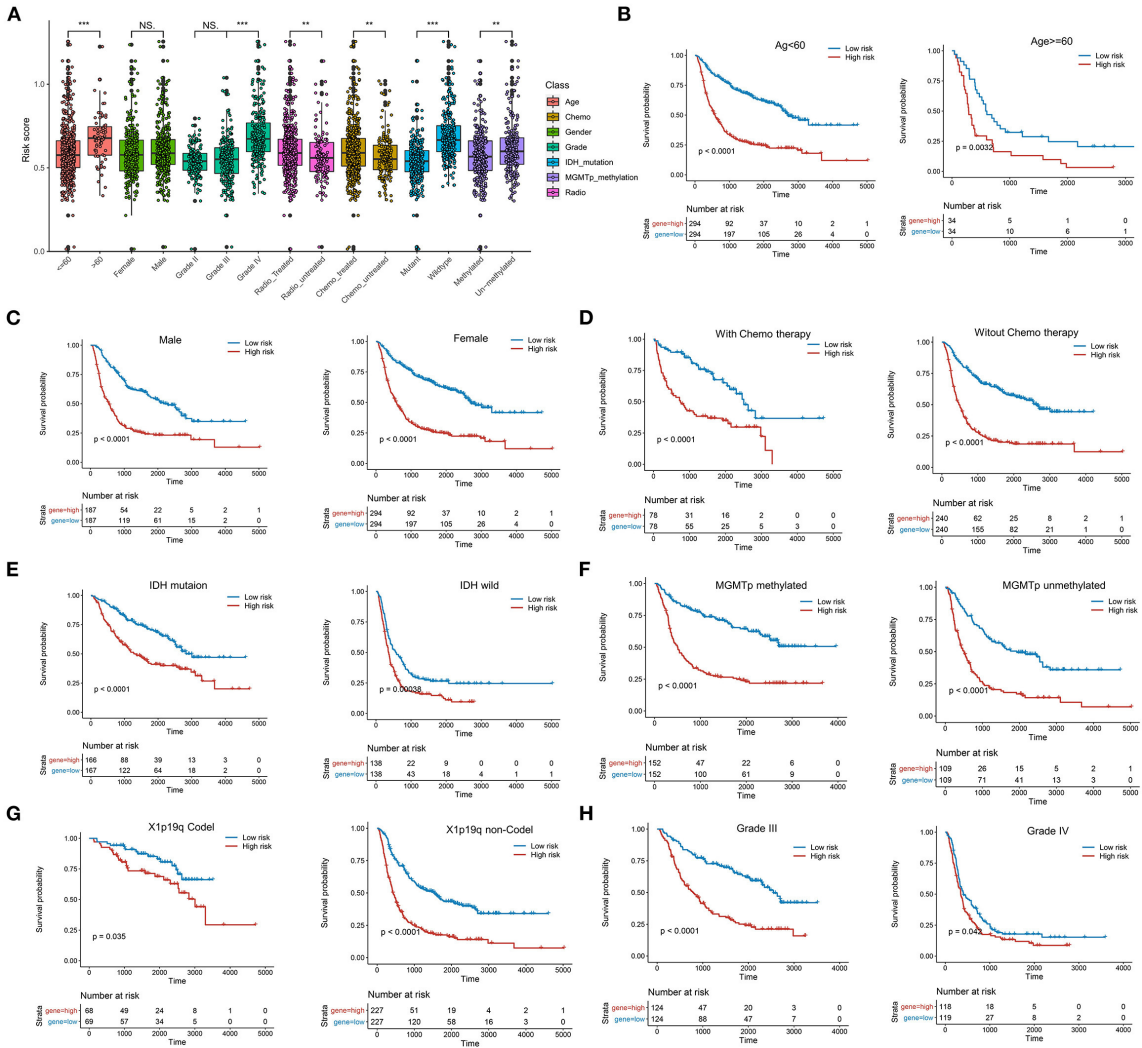
Systematic dissection of LMG signature and clinical parameters in patients with glioma. **(A)** The boxplots illustrating the correlation between LMG signature and different clinicopathological characteristics of patients with glioma in the CGGA-693 cohort. **(B–H)** Kaplan–Meier analysis for OS in different clinical subtypes including age **(B)**, gender **(C)**, chemotherapy **(D)**, IDH mutation **(E)**, MGMTp methylation **(F)**, 1p19q **(G)**, and tumor grade in the CGGA-693 cohort.

We further compared the risk score between patients with different clinical characteristics and found that the LMG signature was associated with age, tumor grade, IDH mutation, radiotherapy, and the O-methylguanine-DNA-methyltransferase (MGMT) status (Figure 4A). However, the LMG signature was not associated with gender (Figure 4A). To further identify the role of the LMG signature for clinical subgroups, patients in the training cohort were divided into subgroups according to the clinical features, including the age, sex, IDH status, 1p/19q status, and WHO classification of the CGGA-693 cohort to compare the survival curves between the subgroup based on the median risk score. The results showed that the LMG signature also had prognostic value in each subgroup, except for patients with grade II in the training cohort (Figures 4B–H).

### 3.5. Construction and validation of the nomogram

**Figure 5:**
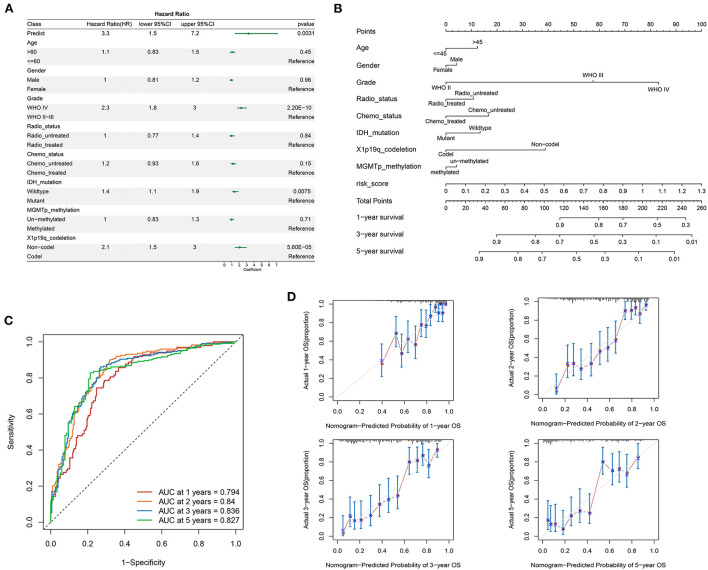
Construction of the prognostic nomogram containing LMG signature in the training set. **(A)** Multivariate cox regression analysis of the clinical parameters and prognostic model for the overall status. **(B)** Nomogram for predicting the survival of patients with glioma. **(C)** Time-dependent ROC curves comparing prognostic accuracy of the nomogram model in the CGGA-693 cohort. **(D)** The calibration curves for predicting the 1-, 2-, 3-, and 5-year OS of the nomogram.

To test whether the LMG signature was an independent predictor for glioma, multivariate analysis was performed using a cox proportional hazards model with the predictive risk score and clinical factors. The results showed that IDH mutation, 1p19q codel, and the LMG-based risk score were independent prognostic predictors of OS (Figure 5A). To evaluate the clinical application of the LMG signature, we constructed a nomogram model by combining the clinical features (age, sex, and neoadjuvant) with the risk score of patients with glioma (Figure 5B). The nomogram showed a favorable predictive ability for OS rates, with a high AUC value of 0.894 (Figure 5C). The calibration curve showed that the predictions of our nomogram model were in good agreement with the 1-, 2-, 3-, and 5-year actual observations (Figure 5D). Thus, our nomogram was more accurate and clinically valuable than any single independent recurrence factor.

### 3.6. Relationship between the LMG signature and TME

**Figure 6:**
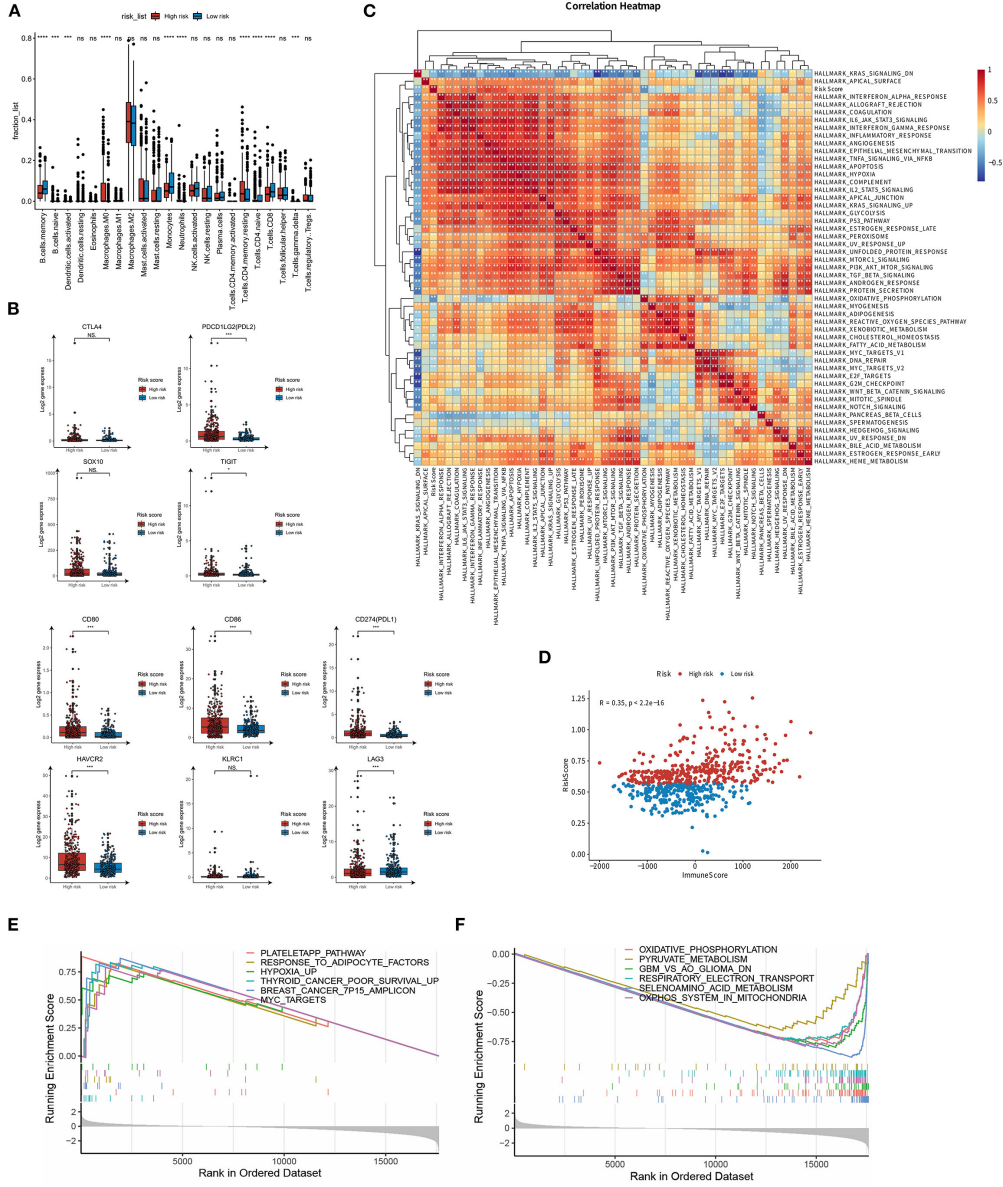
Potential implications for immune infiltration and immune checkpoint gene expression between high- and low-risk LMG signature groups. **(A)** Box plots to display the proportions of 22 immune infiltrating cells in patients with glioma. **(B)** Box plots to show the expression levels of immune checkpoint genes in high- and low-risk LMG signature groups. **(C)** Heat map showing the correlation between the risk score and hallmark gene sets. **(D)** Correlation analysis of immune scores in patients with glioma. **(E, F)** GSEA analysis showing the enriched pathways in the high- **(E)** and low-risk **(F)** LMG signature groups. *p < 0.05, ***p < 0.001.

To evaluate the relationship between these TME indicators and the LMG signature, we used the CIBERSORT algorithm (19) to calculate the proportion of the tumor-infiltrating immune cells in the TME in patients with glioma. The proportion of M0 macrophages, monocytes, CD4^+^ T memory resting cells, and Treg cells was high and that of memory B cells, NK cells, and CD8^+^ T cells was low in patients of the high-risk group (Figure 6A), suggesting a lack of the cell killing activity within tumors of patients in the high-risk group, most probably due to the reduced number of NK cells and less CD8^+^T infiltration. Meanwhile, we compared the expression level of immune-checkpoint genes between the low- and high-risk LMG signature groups and found that CD80, CD86, PDL1, PDL2, HAVCR2, and TIGIT were highly expressed in the high-risk groups (Figure 6B). The upregulation of PDL1, PDL2, HAVCR2, and TIGIT could be in an immunosuppressive status, thus facilitating tumor progression.

### 3.7. Relationship between the LMG signature and cancer hallmark pathways

To evaluate the relationship between the LMG signature and cancer hallmark pathways, we collected the hallmark gene set from the MsigDB dataset and calculated the pathway activity in each sample using GSVA (14). The GSVA scores of these samples were then subjected to Spearman correlation analysis with the risk coefficients of the samples. It was found that the risk score was highly correlated with the “IL6 JAK STAT3 pathway,” “epithelial-mesenchymal response,” and “TNF-α signaling *via* NFKB” pathway (Figure 6C). We further used ESTIMATE (20) to calculate the immune score of each patient and then analyzed the correlation between the risk score and the immune score. It was found that there was a significant positive correlation between the risk score and the immune score, and the *r*-value calculated by Pearson was 0.35 (Figure 6D). GSEA was further performed to identify the involvement of pathways regulating tumorigenesis in the high-risk group. We found that multiple classic tumor-related pathways were enriched in the high-risk group, such as “hypoxia,” “MYC targets,” and “cancer poor survival” pathways (Figure 6E). In contrast, metabolism-related pathways were enriched in the low-risk group, including “Oxidative phosphorylation,” “selenoamino acid metabolism,” and “pyruvate metabolism” pathways (Figure 6F). These results indicate that the LMG signature was associated with the immune response and tumorigenesis pathway. Lactic acid metabolism may affect immune cell function to affect glioma progression.

### 3.8. The underlying genetic alteration of LMGs

**Figure 7:**
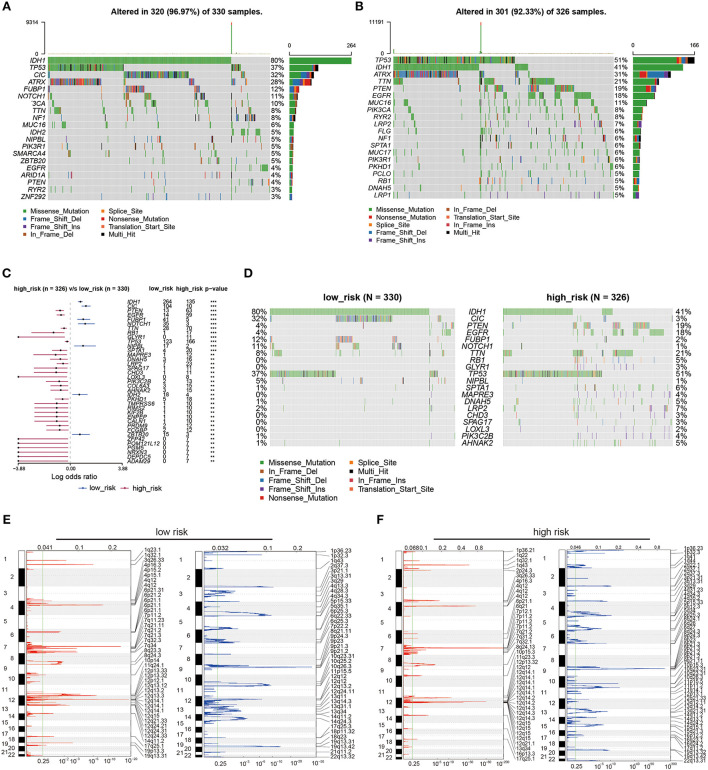
Genetic alterations between high- and low-risk LMG signature groups. **(A, B)** Waterfall plots showing the top 20 mutation landscapes of the low- **(A)** and high-risk **(B)** LMG groups in the TCGA dataset. **(C)** Forest plot showing the significantly different mutated genes between the low- and high-risk LMG groups. **(D)** Waterfall plots showing the significantly different mutated genes between the low- and high-risk LMG signature groups. **(E, F)** The distribution of copy number variation (CNV) features across all chromosomes for the low- **(E)** and high-risk **(F)** LMG signature groups.

To investigate the underlying genetic alteration of the risk level defined by LMG signature in glioma, we obtained TCGA-LGG/GBM somatic mutation profiles and analyzed the mutation landscape for patients with high- and low-risk (Figures 7A, B). We explored the top 20 mutated genes in two groups, respectively. The gene with the most mutation frequency was IDH1 (80%) in the low-risk group and that in the high-risk group was TP53 (51%). We also calculated the significant differentially mutated genes between the two risk groups. As shown in Figures 7C, D, IHD1, CIC, FUBP1, and NOTCH1 were found with a much higher mutation rate in the low-risk group than in the high-risk group. In contrast, PTEN, EGFR, TTN, and RB1 showed a much lower mutation rate in the low-risk group than in the high-risk group. Additionally, the CNV alteration landscapes of the high- and low-risk groups were further compared. The two groups had distinct CNV alteration profiles (Figures 7E, F). The above results suggested that genetic alteration may affect the expression of LMG, which further affect tumor progression.

### 3.9. Prediction of the chemotherapy response by LMGs

**Figure 8:**
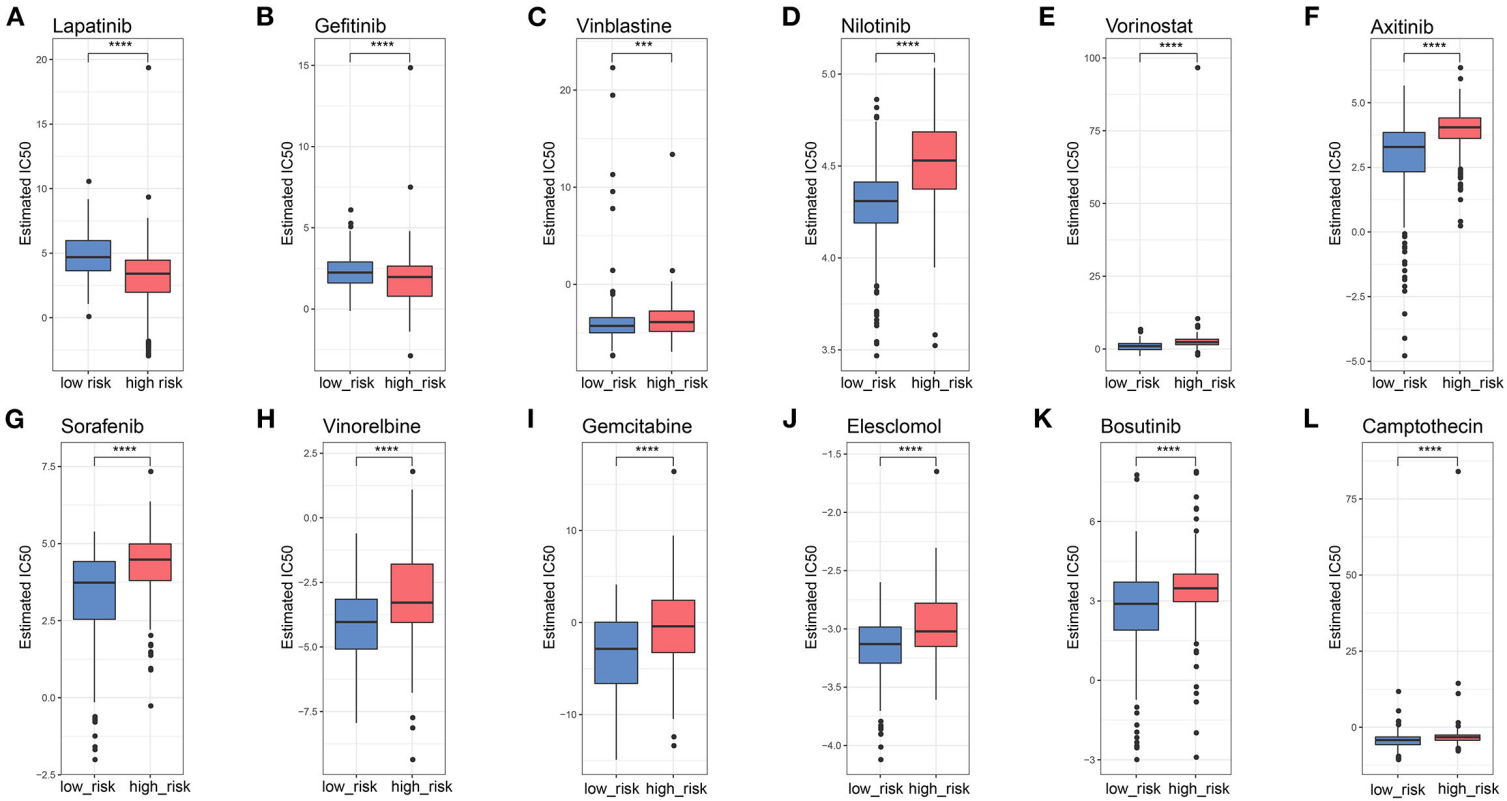
Potential candidate drugs using GDSC analysis. **(A–L)** Boxplots showing the IC50 values of chemotherapy agents for high- and low-risk LMG signature groups. ***p < 0.001, ****p < 0.0001.

To explore potential therapeutic agents for patients with glioma, GDSC analysis was used to predict the chemotherapy response between the two groups (Figures 8A–L). Several chemotherapeutic agents were significantly different between high- and low-risk groups with regard to their sensitivity. EGFR inhibitor Lapatinib and Gefitinib had higher IC50 in the low-risk group than that in the high-risk group (Figures 8A, B), suggesting EGFR inhibitors could be potential pharmacological therapies for patients with high-risk glioma. Other chemotherapeutic agents, such as Vinblastine, Nilotinib, Vorinostat, Axitinib, and Sorafenib also had distinct sensitivity between the two risk groups. The IC50 values of these agents were significantly higher in patients with high-risk glioma than the patients with low-risk glioma (Figures 8C–L), suggesting that patients with high-risk glioma may have tolerance for these drugs and ultimately be able to resist.

## 4. Discussion

Despite considerable advances in the diagnosis and treatment of glioma, it remains cancer with high morbidity and mortality. Increasing evidence suggests that metabolic changes in tumors can modify their microenvironment, and then the newly remodeled microenvironment confers an advantage to tumor cells (21). However, no research has been published on a signature as a prognostic indicator in glioma. In the present study, we generated an LMG-based signature on a training cohort and two independent validation cohorts to predict the survival of patients with glioma. We found that patients in the high-risk group had shorter OS with a high average AUC >0.729. The lactate metabolism signature was also correlated with clinical features, immune infiltration, the expression of checkpoint molecules genetic alteration, activated pathways, and drug sensitivity. These findings illustrate that the novel LMG signature had strong predictive power in OS prediction, suggesting that lactate metabolism may affect tumor progression.

Based on LMGs, we constructed a prognostic risk model to predict the prognosis of patients with glioma. The result showed that our model had good performance in predicting survival state in both the training and validation cohorts. The LMG signature was composed of five crucial genes, including LDHA, LDHB, MRS2, SLC16A1, and SLC25A12 among which LDHA is responsible for the conversion of pyruvate into lactate and NAD+, while LDHB is responsible for converting lactate into pyruvate, fuelling oxidative metabolism (7). The inhibition of MAPK by suppressing LDHA activity decreases the production of pro-inflammatory cytokines in macrophages (22). The LDH inhibitor also reduces the ATP levels and induces oxidative stress and cell death in tumor cells (23). Several studies have shown that lactate and LDHA contribute to tumor progression (24). The downregulation of LDHB is an early event in the development of breast, prostate, and pancreatic cancer (25–27) and is correlated with high proliferation, increased tumor cell invasion, and unfavorable survival outcomes (28). MRS2 is involved in mMg 2+ uptake machinery *via* lactate-mediated conductance (29). Increasing MRS2 levels suppress gastric cancer cell apoptosis and reduce levels of MRS2, leading to abnormal mitochondrial function and decreased mitochondrial ΔΨ (30). SLC16A1 is responsible for transporting monocarboxylic acid metabolites. SLC16A1 plays a crucial role in cancer metabolism (31). Lactate maintains glycolysis efficiency by regulating the pH level in cells and interstitium and upregulating SLC16A1 expression. Cancer cells can increase carcinogenicity and invasion by overexpressing SLC16A1 and transporting lactic acid inward. Targeting SLC16A1 may, therefore, prove to be a promising therapeutic strategy for some cancers (32, 33). The SLC25A12 gene encodes a calcium-binding mitochondrial carrier protein involved in the exchange of aspartate for glutamate across the inner mitochondrial membrane. Loss of SLC25A12 impairs the cytosolic aspartate levels, NAD+/NADH ratio, mitochondrial respiration, and tumor growth (34). Our study showed that LDHA, MRS2, and SLC16A1 were indicators of poor survival, while LDHB and SLC25A12 were indicators of favorable prognosis, suggesting that lactate metabolism is dysregulated in glioma and this dysregulated expression is closely related to tumor progression.

Hypoxia and abnormal metabolite levels, specifically lactate, often characterize disordered metabolic states that contribute to the immunosuppression of the TME. Excessive lactate secreted by metabolism-reprogrammed cancer cells regulates immune responses *via* causing extracellular acidification, acting as an energy source by shuttling between different cell populations, and inhibiting the mTOR pathway in immune cells (35). We found that there were great differences in the immune microenvironment between high- and low-risk groups, showing that the abundance of CD4^+^ T cells, CD8^+^ T cells, and B cells was relatively low in the high-risk group. It was reported that CD8^+^ T cells activate cytolytic activity and effector function, which in turn enhances antitumor responses (36). CD4^+^ T cell and B cell also help and innate signals to DC functions and CD8^+^ T cell priming, enhancing the antitumor immunity (37). Furthermore, in addition, we found that the expression of PDL1, PDL2, HAVCR2, and TIGIT was elevated in the high-risk group. There is evidence that lactate-induced expression of GPR81 induces tumors to express immune checkpoint ligand PD-L1 (38), indicating that lactate-mediated immune dysregulation can distort host immune checkpoints in various ways to escape immune responses and promote the development and progression of glioma. Our findings are consistent with other studies showing that increased LDH activity triggers tumor immune escape by inhibiting immune function (39, 40). Although tumor immunotherapy has demonstrated good therapeutic outcomes in some cancers, few studies have reported its efficacy in gliomas (41). Our LMG-based model may be useful for screening patients for glioma immunotherapy.

Our study illustrated that patients with glioma in the two risk groups had distinct genetic alterations, activated pathways, and potentially sensitive drugs. A common characteristic of gliomas is the presence of markers for IDH1 and TP53 mutations, which influence the fate of the cells (42). The two risk groups were IDH1- and TP53-dominant mutations, respectively, suggesting discrepant oncogenesis mechanisms. These two mutations could be the cause of metabolism disorders. Recent studies reported that the IDH1 mutant glioma tissue displayed massive alterations in glycolysis and lipid metabolism compared with IDH1 wild-type glioma tissue. Both groups showed similar levels of tricarboxylic acid (TCA) cycle intermediates, but IDH1 mutant gliomas accumulated more pyruvate (43). TP53 can affect glycolysis and mitochondrial oxidative phosphorylation pathways and promote tumor development (44). Furthermore, the high-risk group had increased mutations of PTEN and EGFR. PTEN occurs in ~20% of glioblastomas but is rare in lower-grade gliomas, suggesting PTEN suppressor genes involved in the development of glioblastomas (45). There have been several EGFR gene alterations identified in gliomas, especially in glioblastomas, which acted as a prognostic factor and a predictor of treatment response in patients with glioma (46). Thus, EGFR inhibitors were predicted to be potential pharmacological therapies for high-risk groups in patients with glioma. Our study showed that the LMGs may have cross-talks with pro-oncogenic signaling pathways or other metabolism pathways. MYC could act as a glycolysis regulator and modulate both glucose transport and glucose breakdown into lactate by targeting genes (47). The activated MYC targets in high-risk groups could be a potential way to promote tumorigenesis. The other metabolism pathways, such as oxidative phosphorylation, were also reported to affect invasion ability, drug sensitivity, and prognosis (4, 48). Through drug sensitivity analysis, we found that the two LMG risk groups had distinct sensitivity to chemotherapy drugs. Our model may help to develop individualized treatment for patients with glioma.

In addition, our multivariate cox model showed that our LMG model remained independent of other factors such as IDH mutation and X1p19q deletion for patients with glioma. Using routine clinical factors associated with OS, we developed a nomogram model for clinical applications. The calibration plot showed good consistency between the prediction by nomogram and actual observation of the survival in glioma, suggesting that the proposed nomogram model could be used as a supportive tool to help clinicians distinguish, assess, and evaluate the risk and prognosis of patients with glioma.

In conclusion, we comprehensively explored the characteristics of LMGs in glioma and established a novel prognostic model to stratify different risk groups of patients with glioma, which may help plan individualized treatment and improve clinical survival outcomes of patients with glioma.

## Data availability statement

The original contributions presented in the study are included in the article/Supplementary material, further inquiries can be directed to the corresponding authors.

## Ethics statement

Ethical approval was granted by the Ethics Committee of Shanghai Changhai Hospital (Ethics approval number: CHEC2020-164). The patients/participants provided their written informed consent to participate in this study. Written informed consent was obtained from the individual(s) for the publication of any potentially identifiable images or data included in this article.

## Author contributions

WY, ZK, and JL designed the overall research strategy. ZW, JW, ZK, and YL performed the bioinformatics analysis and drafted and revised the manuscript. JL and WY participated in the data discussion. All authors contributed to this manuscript and approved the submitted version.

## Supplementary material

Supplementary Figure 1 Immunohistochemistry staining of lactic acid metabolism-related genes (LMGs). **(A–L)** Immunohistochemical (IHC) staining of LDHA **(A, B)**, LDHB **(C, D)**, SLC25A12 **(E, F)**, HIA1A **(G, H)**, MRS2 **(I, J)**, and SLC16A **(K, L)** retrieved from Human Protein Atlas.

Supplementary Table 1 Clinical information in the included datasets.

Supplementary Table 2 The primer sequence of the six LMG-genes for the qRT-PCR.
